# Selection of Diethylstilbestrol-Specific Single-Chain Antibodies from a Non-Immunized Mouse Ribosome Display Library

**DOI:** 10.1371/journal.pone.0033186

**Published:** 2012-03-13

**Authors:** Yanan Sun, Baoan Ning, Ming Liu, Xianjun Gao, Xianjun Fan, Jianqing Liu, Zhixian Gao

**Affiliations:** 1 Tianjin Key Laboratory of Risk Assessment and Control Technology for Environment & Food Safety, Institute of Hygiene and Environmental Medicine, Academy of Military Medical Sciences, Tianjin, China; 2 Department of Biochemistry and Molecular Biology, Tianjin Medical University, Tianjin, China; 3 Research Center of Basic Medical Sciences, Tianjin Medical University, Tianjin, China; 4 School of Chemical Engineering and Technology, Tian Jin University, Tianjin, China; University of Kentucky, United States of America

## Abstract

Single chain variable fragments (scFvs) against diethylstilbestrol (DES) were selected from the splenocytes of non-immunized mice by ribosome display technology. A naive library was constructed and engineered to allow in vitro transcription and translation using an *E. coli* lysate system. Alternating selection in solution and immobilization in microtiter wells was used to pan mRNA-ribosome-antibody (ARM) complexes. After seven rounds of ribosome display, the expression vector pTIG-TRX containing the selected specific scFv DNAs were transformed into *Escherichia coli* BL21 (DE3) for expression. Twenty-six positive clones were screened and five clones had high antibody affinity and specificity to DES as evidenced by indirect competitive ELISA. Sequence analysis showed that these five DES-specific scFvs had different amino acid sequences, but the CDRs were highly similar. Surface plasmon resonance (SPR) analysis was used to determine binding kinetics of one clone (30-1). The measured K_D_ was 3.79 µM. These results indicate that ribosome display technology can be used to efficiently isolate hapten-specific antibody (Ab) fragments from a naive library; this study provides a methodological framework for the development of novel immunoassays for multiple environmental pollutants with low molecular weight detection using recombinant antibodies.

## Introduction

Diethylstilbestrol (DES) is a synthetic nonsteroidal estrogen that was first synthesized in 1938. Initially, DES was used clinically to prevent spontaneous abortion. However, follow-up studies indicated that DES also has the potential to cause a variety of significant adverse medical complications during the lifetime of those exposed. The long-term consequences of DES exposure to women include increased risk of breast cancer and cervicovaginal cancer. Abnormalities in offspring have also been reported, including immune system disorders, psychosexual effects, and reproductive abnormalities [1]. Moreover, DES has also been employed as a growth-promoting agent to accelerate weight gain and improve feeding efficiency in cattle. Use of growth-promoting drugs for fattening livestock has been banned in the European Union since 1986 to protect consumers from possible harmful effects from the intake of estrogen residues. Food toxicology studies have reported that the illegal application of DES as a growth promoter is still widespread, and has become a significant hazard to human health [2]. This has given rise to the need for a sensitive assay to detect DES in environmental samples. Immunoassays are now widely used for this purpose due to their high specificity and sensitivity [3]. The prerequisite preparation of anti-hapten antibodies by animal immunization is time consuming and difficult, however. In addition, different species of animals may have different immune responses to haptens, and in some cases, no immune response may be evoked.

Recently, display technologies have provided powerful and efficient approaches for the development and selection of antibodies in vitro [4]. It is well known that phage display for antibody fragments represents a considerable advance compared to hybridoma technology, and is now widely used for the selection of antibody fragments. Since the development of phage-display technology, several other means of displaying antibodies have been proposed, such as display on ribosomes, yeast, and bacterial cells. Compared to phage display, ribosome display bypasses the deficiencies inherent in the development and selection of antibodies by phage display. First, ribosomal display avoids the transformation limits in phage display, as the size of a ribosome display is potentially very large and diverse, which enhances the opportunity for selecting the highest affinity and specificity antibodies. Furthermore, the transcription, translation, and panning are performing in cell-free system, which allows for the expression of toxic proteins and circumvents random mutations introduced by PCR [5]. Ribosome display technology has been successfully used for the selection and evolution of ligand-binding proteins [6], [7], enzymes [8], and other peptides [6], [9]. However, reports on selection of antibodies against haptens by ribosome display are scarce. Human anti-progesterone single-chain fragments were isolated from a transgenic mouse library [10]. Progesterone-binding fragments were selected over five cycles of ARM display and expressed in *E. coli* [10]. The affinity of the expressed antibody fragments was 10^−8^ M [10]. In addition, scFvs were selected from hybridoma cell lines against sulfadimidine (SM_2_) by using the ribosome library technology. Three positive clones (SAS14, SAS68, and SAS71) were screened from 100 clones and all had high antibody activity and specificity to SM_2_ [11].

On the other hand, a naive library is created from genetic material that has never been exposed to antigen prior to library construction. Although higher-affinity binders can be selected from immune libraries, immunizations are generally required for each targeted antigen, which hampers high-throughput. In addition, the immunization of small molecules conjugated to carrier protein may create uncertainty regarding the true scFv target. Currently, a number of anti-haptens have been isolated from naive library using phage display, such as steroids [12], fluorescein [13], phenyl-oxazolone [14], and mycotoxin [15]. Human scFv antibodies specific for the haptens digoxigenin, progesterone, testosterone, and estradiol have been isolated from a naive IgM library [12], thereby demonstrating the feasibility of naive libraries for isolating antibodies. Naive libraries are ready to use for any antigen without immunization. The size of the library must be sufficiently large to obtain high-affinity binders, however, a condition not met by phage display.

Thus, we chose the naive library for selecting anti-DES antibodies by ribosome display technology. Currently, there have been few reports on selecting haptens from a naive library using ribosome display. A naïve llama ribosome display library has been used to select single-domain antibodies against the hapten picogram [16]. In our study, we have constructed a naive library of scFvs from the splenocytes of non-immunized mice, and DES-specific scFvs were selected from the library using ribosome display technology. The new selection strategy of alternating selection between solution and microtiter wells was applied to isolate DES-specific scFvs from the library while eliminating non-specific binders. We used the expression vector pTIG-TRX with the molecular chaperone thioredoxin to facilitate soluble expression and obtain soluble scFvs from the cytoplasmic supernatant. We further describe an indirect competitive assay to characterize the binding properties of the isolated scFv antibodies specific to DES. Surface plasmon resonance analysis was used to determine binding kinetics. The aim of this study is to provide a method to isolate recombinant antibodies against low molecular weight molecules (haptens) such as drugs of abuse, vitamins, hormones, metabolites, food toxins and environmental pollutants.

## Results

### Construction of the naive ribosome display library

**Figure 1:**
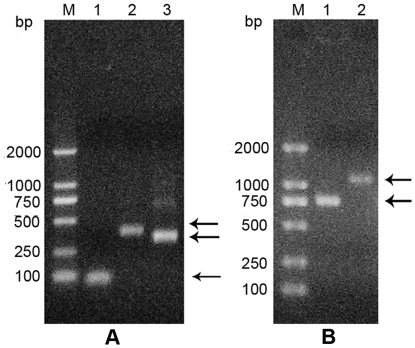
Agarose gel electrophoresis of amplified GS, VH,VL, scFv DNA fragment and assembled naive library. Arrows indicate DNA fragments positions. (A) Lane M: D2000 DNA marker, lane 1: GS fragments, lane 2: VH fragments, lane 3: VL fragments. (B) Lane M: D2000 DNA marker, lane 1: Assembled scFv DNA fragment, lane 2: 1.2 kb assembled naive library.

The naive scFv library was prepared from non-immunized female mice. Total RNA was extracted from spleen cells of non-immunized mice with TRIZOL according to the manufacture's instructions (Invitrogen, USA) and a cDNA library was constructed by reverse transcription. The VH and VL fragments were amplified by RT-PCR from the cDNA library. The amplified VH and VL fragments were the expected size (Figure 1). In order to avoid incorrect assembly, a 81 bp GS DNA fragment with ends complementary to the downstream sequence of the VH fragment and the upstream sequence of the VL fragment was synthesized. The assembled full-length scFv fragments (approximate 1.2 kb) were used for the construction of templates for ribosome display (Figure 1). The assembled 1.2 kb scFv DNAs contained T7 promoters, ribosome binding sites and spacer proteins. Therefore, the assembled library was used for preparation of mRNA in vitro.

### The sequences analysis of scFv library and ribosome display whole library

**Figure 2:**
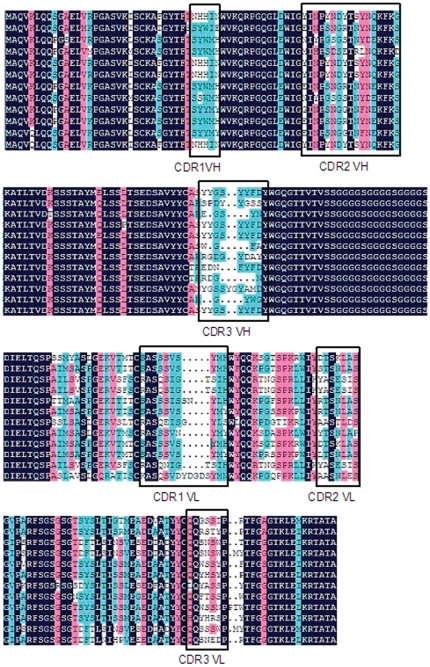
The deduced amino acid sequences of the scFvs and complementary determining regions (CDRs). The deleted residues are represented by a dash (-). FRs and CDRs are determined according to the Kabat database.

To assess the integrity of the library, a fraction of the scFv library and whole assembled library were subcloned into a plasmid vector. The scFv library DNA of 15 individual clones and the assembled whole library DNA of 13 individual clones were sequenced. Twelve sequences from 15 individual clones were correctly in reading frame and had no stop codon. The deduced amino acid sequences of the scFv library sequences and complementary determining regions (CDRs) are shown in Figure 2. The FRs and CDRs were determined according to the Kabat database. The diversity of the scFv library was also examined by direct DNA sequencing. There were five amino acid residues in CDR1 VH and seventeen amino acid residues in CDR2 VH. The length of CDR3s ranged from 5 to 11 amino acid residues, with an average length of 8 residues in CDR3 VH. There were 7 amino acid residues in CDR2 VL and CDR3 VL. The length of CDR1 VL was variable. This diversity in the amino acid sequences of all three CDRs indicating that scFvs of various binding characteristic should be available in the library. Nucleotide sequence data indicated that 9 sequences from 13 individual clones for in vitro transcription and translation were incorporated in frame within the scFv sequences and spacer sequences. In addition, no stop codon was detected in any of the reading frames. The whole library was then prepared for transcription and translation in vitro.

### The affinity panning of scFvs by ribosome display

**Figure 3:**
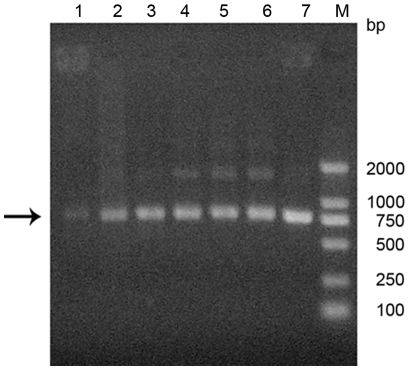
Selection and amplification of anti-DES scFv gene over seven rounds of ribosome display. After selection, the eluted RNA was amplified by RT-PCR and the products were analyzed by agarose gel electrophoresis. Lane M: D2000 DNA marker, lane 1–7: recovered band from the first to seventh selected library of ribosome display. Arrow indicates scFv DNA fragments position.

The scFv library DNA which was placed in the context of the *E. coli* ribosome-display format was subjected to in vitro transcription and translation using *E. coli* S30 extract lysate to generate ternary AMR complexes. Selections can be performed either with ligands immobilized on a plastic surface or in solution [17]. To avoid the selection of binders specific to the carrier proteins, antigen swapping was incorporated into the selection protocol. DES-BSA and DES-coupled magnetic beads were used in turn in the selection. For the fifth and seventh round of selection, DES-coupled magnetic beads were used, while DES-BSA was used in the other rounds of selection. By the end of the first round, only a weak DNA band was visible. The quantity of RT-PCR products continually increased during subsequent rounds of panning. Based on the result of RT-PCR, enrichment of specific scFvs was clearly confirmed (Figure 3).

### Soluble expression of scFvs

**Figure 4:**
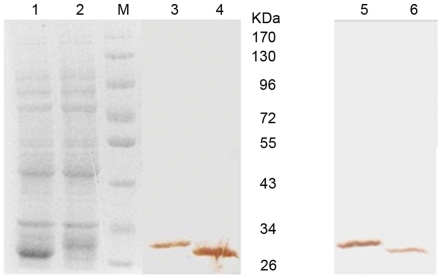
SDS-PAGE assay and immunoblot analysis of the two random selected soluble anti-DES scFvs (approximately 30 kDa). Lane M: protein standards; lane 1, 2: SDS–PAGE analysis of the induced whole cellular lysate; lane 3, 4: Western blotting analysis of the induced whole cellular lysate; lane5, 6: Western blotting analysis of cellular supernatant.

**Figure 5:**
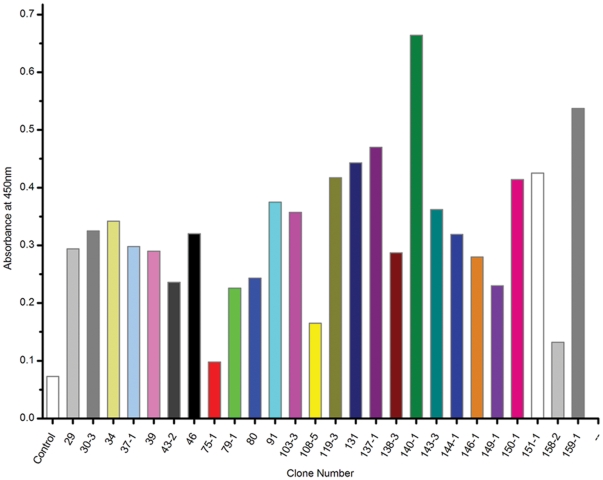
Binding activity of soluble scFvs to DES-OVA by indirect ELISA. The background (using wells coated with OVA) was subtracted from the absorbance of each DES conjugate. Control was the supernatant of the *E. coli* BL21 (DE3) without scFv gene.

The scFv library selected after the seventh round was inserted into the cloning vector pMD18-T and transformed into DH5α. The seventh round scFv library DNA of 100 individual clones was sequenced. Forty-three individual clones had no stop codon and were in correct reading frame, and these were used to express scFv proteins. Of these clones, 26 of 43 selected after the seventh round were inserted into the expression vector pTIG-TRX, and then transformed into the expression bacteria BL21 (DE3). Supernatants were extracted from each colony to verify the location and functionality of each soluble scFv. The expression of each scFv was confirmed by SDS-PAGE and Western blot analysis, and the expressed supernatants of two individual clones selected randomly were identified by Western blot analysis using anti-his tag antibodies (Figure 4). Proteins of approximately 30 kDa in size were expressed from these two randomly selected clones. However, because of the different length of CDR3 VH and CDR1 VL, slight differences existed in the size of the expressed proteins. The binding activity to the antigen of 26 expressed scFv supernatants was measured by indirect ELISA assay (Figure 5). Results suggested that all of the soluble scFvs exhibited significant binding to DES except for 75-1 and 158-2. These results confirmed that the expression vector pTIG-TRX with the molecular chaperone thioredoxin was able to express the soluble protein effectively.

### Characterization of soluble scFvs

**Figure 6:**
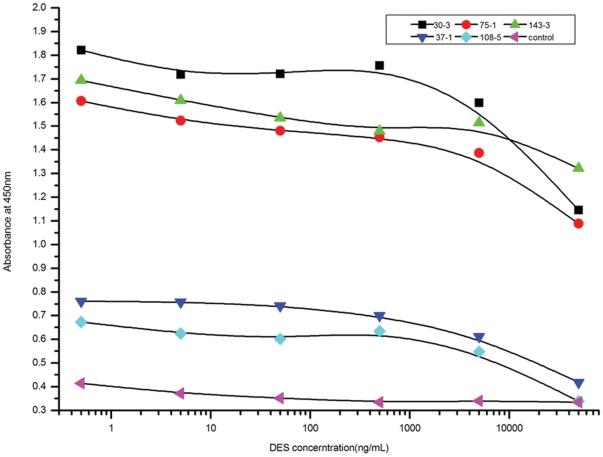
Competition assay for scFv to DES. Different concentrations of free DES, ranging from 0.0005 to 50 µg/mL were used to inhibit the binding of the scFvs to DES-OVA conjugate. Control was the supernatant of *E. coli* the BL21 (DE3) with pTIG-TRX without scFv gene.

The 26 crude expressed extracts specific to the DES conjugates were assessed for their specificity to free DES by a competition assay (Figure 6). The binding of each scFv to DES-OVA was inhibited with different concentrations of free DES. Five of the crudely expressed supernatants exhibited obvious competitive inhibition of binding. The absorbances at 450 nm of these five samples decreased with increasing concentrations of free DES. On the other hand, the binding of the scFv antibodies to soluble free DES varied among clones. Five of the scFv antibodies were significantly inhibited from binding to DES–OVA-coated microtiter wells by 50 µg/mL soluble DES. Two antibodies, 30-3 and 75-1, were most inhibited from binding to DES-OVA-coated microtiter wells at 50 µg/mL free DES in solution. ScFv antibodies 143-3, 37-1, and 108-5 were partially inhibited from binding to DES-BSA coated microtiter wells at 50 µg/mL DES. We thus concluded that the ribosome display library was able to screen scFvs specific for DES by alternating selection using BSA and DES-conjugated magnetic beads.

**Figure 7:**
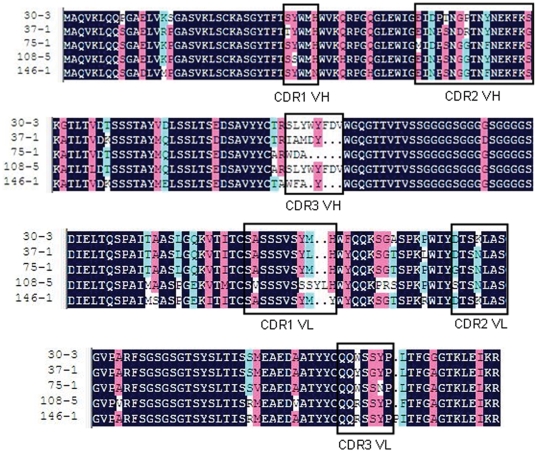
Deduced amino acid sequences of the five different scFvs from the competitive ELISA. The deleted residues are represented by a dash (-). FRs and CDRs were determined according to Kabat database.

The coding sequences of the 5 positive scFv clones were identified (Figure 7). These sequences displayed a high level of homology with each other. There were only one or two different amino acid residues in CDR1, 2 in VH and CDR2, 3 in VL of the five coding sequences. In CDR1 VL and CDR3 VH, there were also differences in the number of amino acid residues, but the result of sequencing suggested that they were homologous from Sequence analysis showed that each DES-specific scFv had a different sequence, but the CDRs of the five positive scFv clones after seven rounds selection were similar. The conserved region in these scFvs may be associated with specific binding to DES.

**Figure 8:**
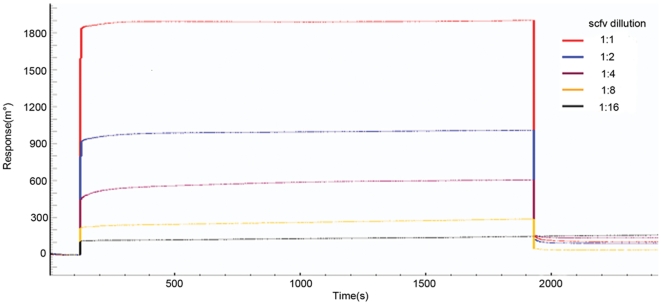
The calculation of association rate constants by SPR. 30-3 scFv was appropriately diluted in PBS at five antibody concentrations.

To calculate the association rate constant, 30-3 scFv was appropriately diluted in PBS and analyzed at several concentrations (Figure 8). The equilibrium dissociation constant (K_D_) for the 30-3 clone was also calculated. Equilibrium dissociation constants (K_D_) were determined independently by Kinetic Evaluation 5.0 software. The K_D_ of 30-3 was estimated to be 3.79 µM.

## Discussion

In our experiment, we successfully have constructed a naive scfv library for transcription and translation in vitro. Samples from the library were cloned into a sequencing vector for their DNA analysis, and there is strong evidence that the library constructed in this study has diverse scFvs sequences. 12 sequences of 15 individual clones were correctly in reading frame and no stop codon. There are five amino acid residues in CDR1 VH and seventeen amino acid residues in CDR2 VH. The length of CDR3s ranges from 5 to 11 amino acid redidues, with an average length of 8 residues in VH. There are 7 amino acid residues in each CDR2 VL and CDR3 VL.The length of CDR1 VL were various. In a word, diverse compositions of all the CDRs may display different binding characteristics of different scFvs in the library. However, in the process of the construct of the library, we found that the times of amplification for VH and VL and the ways of the linkage for V region may induce the site mutation and stop codons. In our preliminary experiment, the lethal mutation and error-frame mutation appeared when using the linker which was not synthesized. The stop codons existed 50% in the unselected library, and then the library had to be discarded completely. The amplification error can be reduced effectively by decreasing the times of PCR and using the high fidelity polymerase for amplification.

After seven rounds of selection, the selected scfv library was sequnenced and the result indicated that 43 individual clones of 100 had no stop codon and were in correct reading-frame, the ratio of mutation reached up to about 57%. In comparison, the ratio of mutation to death for the original scFv library and the fourth round scFv library were 20% and 30%, respectively. Then we deduced that the mutation was accumulated with the increase of the selection round and this was used to perform the maturation of recombinant antibody by ribosome display. In vitro selection cycles enable the introduction of further diversity, providing a means of protein evolution. Thus ribosome display and similar methods are routes to increased diversity, improved selection and a range of potentially novel molecules [18].

In vitro translated mRNA-scFv complexes linked to ribosomes were selected with DES-BSA (i.e. rounds 1, 2, 3, 4 and 6) and DES-coupled magnetic beads (i.e. rounds 5 and 7) to maximize the recovery of hapten-binders over carrier binders. Using the alternated selection in solution and immobilized in the microtitre wells may improve the binding activity of the scFvs and specificity to DES. In addition, there were two advantages in using DES-coupled magnetic beads for selection. Firstly, immobilization of protein antigens on plastic surfaces, however, may lead to partial unfolding of the protein due to hydrophobic interactions with the plastic. In extreme cases, this can lead to the selection of antibodies against protein epitopes that are not accessible in the native form. This can be circumvented by selecting in solution. Secondly, it can subtract the binders specific to the carrier proteins. In our study, the isolated five scFvs performed the specificity to DES according to the result of indirect competitive ELISA. Serials of DES concentration were mixed with the expressed scFvs and then added to the microtitre wells for incubation at 2 h. Obviously, the absorbance at 450 nm was decreased as the increase of DES concentration. Therefore, the results implied that the selected scFvs were specific or partially to the DES. In the indirect ELISA for identifying the binding activity of scFvs after 7 rounds, some individual clones showed high binding. However, some high binding scFvs has the light inhibition using the indirect competitive ELISA. It seems that the high binding activity to DES-protein contains binding to the bridging group linking the protein and the hapten. Furthermore, in biopanning, direct haptens conjugation to ELISA plate wells could be tried. The method may also avoid selecting other binders excepted for the small molecular.

Immune libraries may provide higher-affinity binders, but immunizations are generally required for each targeted antigen, although multi-antigen immunizations have been performed successfully [19]. Naive and synthetic libraries, on the other hand, are ready to use for any antigen without immunization. However, even though several systematic studies have attempted to create hapten-directed recombinant antibodies [20], [21], [22]. In this study, we have firstly selected hapten-specific scFv from a naive library using ribosome-display technology and it demonstrates the availability of selection of multifarious environmental contaminants which are hydrophobic small molecules from a naive library by ribosome display. This method bypasses the immunization of animals multiply and is highthrough and time-saving. We have isolated and characterized anti-DES scFvs. To our knowledge there have been no report on isolating anti-DES scFvs from a naive library using ribosome display. The naive library, tend to have low affinity for their antigen, be it protein or hapten [23]. However, this low affinity may be overcome through the construction of very large libraries comprising up to 10^12^ different antibodies. Such large libraries are tedious to construct using phage display, because of the very high number of transformation events required, but the enough large scFv library can be used in ribosome display technology. The large libraries means the large diversity of the library, and this is paramount to the success of the selection experiments. The DNA library of scFvs was amplified by splice-overlap extension PCR. With the ability to generate up to 10^15^ copies of dsDNA in one PCR, in addition, in the current ribosome-display experiments, no cloning and transformation steps were involved, therefore, the size of the scFv DNA library for this study is expected to be much higher than that of phage-display library [24].

Compared with the scFvs from immune library, antibodies isolated from non-immune libraries may possess lower affinity. However, modified panning strategies used to increase the likelihood of isolating high-affinity hapten binders are: 1 to gradually decrease the coating conjugate concentration during successive rounds of panning [25]; 2 to use the free hapten to elute the hapten-specific antibodies; 3 to use analogues with which the antibodies are preincubated prior to their incubation with the target hapten conjugate; 4 to perform subtractive panning, where the antibodies are incubated with the carrier protein prior to incubation with the hapten–protein conjugate [26]. Subtractive panning should remove any antibody binding to the carrier protein, but not those antibodies binding to the bridging group linking the protein and the hapten. Subtractive panning, or the incubation of phage antibody mixture with carrier protein alone prior to its transfer to the hapten–BSA conjugate in separate wells, was also performed to select for hapten binding and remove BSA binding antibodies [27]. Subtractive panning proved to be successful as the isolated antibodies did not bind significantly to BSA. Subtractive panning has now become a standard procedure for panning with most hapten–protein conjugates. In further research, in order to improve the affinity and specificity of hapten-scFv, we may perform the above measures.

## Materials and Methods

### Materials

All reagents used in the study were commercially available and were of reagent grade or better. *E. coli* S30 Extract System for Linear Templates, Wizard SV gel, and RNase-free DNaseI were obtained from Promega (USA). The pMD18-T vector was obtained from TaKaRa Biotechnology Ltd. (Dalian, China). The RNeasy Mini Cleanup kit was purchased from Qiagen (Germany). TRIZOL and MLV were obtained from Invitrogen (USA). A HisTrap HP metal affinity chromatography was purchased from GE Healthcare (USA). The pTIG-TRX vector and *E. coli* BL21 (DE3) was supplied by the Beijing Institute of Biotechnology (Beijing, China). Synthetic DNAs were obtained from Invitrogen (USA).

### Library construction

A library of scFv DNAs from non-immunized mice was constructed following the protocol developed by Hanes et al. [24] for prokaryotic ribosome display experiments.

### Construction of a naive scFv library

The total RNA was isolated from spleen cells of non-immunized of mice with TRIZOL according to the manufacture's instructions (Invitrogen, USA). Single cDNAs were synthesized by oligo(dT)_15_ primers. The mouse VH and VL chains were amplified by RT-PCR. The primer pairs HF (5′-TATATCCATGGCCCAGGTSMARCTGCAG-3′) and HR (5′-TGAGGAGACGGTGACCGTGGTGCCTTGGCCCC-3′) were used for amplification of cDNA encoding the VH chain. The primer pairs LF (5′-GACATCGAGCTCACTCAGTCTCCA-3′) and LR (5′-CGCGGTTGCGGTCCGTTTBAKYTCCARCTTKGTSCC-3′) were used for amplification of cDNA encoding the VL chain. A 81-bp DNA linker containing a sequence encoding (Gly_4_Ser)_3_ was amplified with primers GSF (5′-ACGGTCACCGTCTCCTCA-3′) and GSR (5′-CTGAGTGAGCTCGATGTC-3′) using Taq polymerase by 30 cycles of PCR (30 s at 95°C, 20 s at 50°C, and 30 s at 72°C). After gel purification with Wizard SV GEL kit (Promega, USA), the V-genes and the linker were assembled into full-length scFvs using splicing by the overlap extension (SOE) protocol. Briefly, 15 ng of VH DNA, 20 ng of linker DNA, and 15 ng of VL DNA were mixed with 25 µL of PCR mixture without primers and cycled 15 times (30 s at 94°C, 100 s at 72°C) and then the primers HF and LR were added to the mixture. The mixture was subjected to 20 cycles (94°C for 30 s, 55°C for 30 s, 72°C for 80 s). A portion of this scFv library was cloned into the pMD18-T vector (TaKaRa, Japan) and transformed into calcium-competent *E. coli* DH5α cells. Fifteen randomly chosen clones were then sequenced.

### The construction of the clone vector pMD18-t+T and pMD18-t+fPD

The scFv library was used as the starting material in a series of sequential PCRs, which added various elements for efficient in vitro transcription, translation, and folding. The plasmid pT7PD was used to generate two DNA sequences, the T sequence and the fPD sequence by PCR amplification. The T fragment was used to introduce the T7 promoter and the RBS sequence added for efficient in vitro transcription and translation. The PCR protocol consisted of an initial denaturation step at 94°C for 5 min followed by 30 cycles of 94°C for 30 s, 49°C for 30 s, and 72°C for 30 s, then and a final extension step at 72°C for 10 min. The fPD, consisting of amino acids T20-V109 of protein D (fPD), a structured part of the capsid protein from phage Lambda, was used as a spacer sequence [28], [29]. The spacer sequence provides sufficient distance between the displayed protein and the ribosome, allowing the protein to fold into its correct conformation. The PCR protocol consisted of an initial denaturation step at 94°C for 5 min, then 30 cycles of 94°C for 30 s and 70°C for 1 min, followed by a final extension step at 72°C for 10 min. The PCR product was purified and separated by agarose gel electrophoresis. The purified DNA fragments were cloned into the pMD18-T vector (TaKaRa, Japan) and transformed into calcium-competent *E. coli* DH5α cells. Three randomly chosen clones were then sequenced.

### Generation of the naive ribosome display library

The T7-promoter, ribosome binding sites and spacer sequence were amplified from the vector pMD18-T with the right sequences. The T-fragment, fP-fragment, and scFv library were assembled by SOE protocols. The process of assembling the whole sequence was performed in two steps. Firstly, the T-fragment and scFv library were assembled. In the SOE experiment, the reaction mixture (50 ng scFv library genes, 50 ng T sequence, 2×PCR master mixture) was subjected to an initial denaturation step at 94°C for 5 min, followed by 15 cycles of 94°C for 30 s, 44°C for 30 s, and 72°C for 2 min. To amplify the assembled fragment, 25 pmol of each of the two primers T7F (5′-CGCATACGAAATTAATACGACTCAC-3′) and LR were added at cycle 11 and the amplification step was an initial denaturation at 94°C for 5 min, then 8 cycles of 94°C for 30 s, 64°C for 30 s, and 72°C for 2 min, followed by 15 cycles of 94°C for 30 s, 45°C for 30 s, 72°C for 2 min, and a final extension step at 72°C for 7 min. Then, the spacer sequence was added to the newly assembled DNA library. Briefly, 50 ng new assembled library genes, 50 ng fPD sequence, 12.5 µL 2×primerSTR GC buffer, dNTP mixture, and primeSTAR HS DNA polymerase were combined. The thermocycle protocol consisted of an initial denaturation step at 98°C for 2 min, followed by 15 cycles of 98°C for 10 s, 57°C for 5 s, and 72°C for 90 s. To amplify the assembled fragments, 25 pmol of each of the two primers T7F and PDRS (5′-CCGCACACCAGTAAGGTG-3′) were added at cycle 11 and the amplification step was an initial denaturation step at 98°C for 1 min followed by 15 cycles of 98°C for 10 s, 45°C for 5 s, and 72°C for 90 s, followed by a final extension step at 72°C for 5 min. The whole library was amplified in a 50 µL PCR mixture for 25 cycles with T7F and PDR (5′-CCGCACACCAGTAAGGTGTGCGGTAACGATGCTGATTGCCGTTCCG-3′). Then the sequences were purified by a DNA purification kit (Promega, USA). A portion of this whole library was cloned into the pMD18-T vector (TaKaRa, Japan) and transformed into calcium-competent *E. coli* DH5α cells. Thirteen randomly chosen clones were then sequenced.

### In vitro transcription and translation for selection

In vitro transcription and translation reaction was based on *E. coli* S30 lysate (Promega, USA) and performed according to the supplier's protocol. Briefly, 50 µL of transcription/translation mixture containing 5 µL amino acid, 20 µL S30 Premix, 15 µL S30 extract, 2 µL 5 µM anti-ssrA oligonucleotide, and 1 µL RNasin was mixed in a RNase-free tube with 10 µL purified library. After incubation at 37°C for 10 min, the reaction was arrested by a 5-fold dilution with ice-cold WBTH (50 mM Tris-acetate at pH 7.5, 150 mM NaCl, 50 mM magnesium acetate, 500 mM KCl, and 0.5% w/v BSA).

### Affinity selection in microtiter plates

Microtiter plates were coated with 200 µL of DES-BSA solution (100 µg/ml in carbonate buffer solution) or carbonate buffer solution at 4°C overnight. The coated plates were washed with PBS and blocked with sterilized 0.5% (w/v) BSA in PBS for 1 h at 37°C. After washing twice in PBS and twice in WBT (50 mmol/L Tris-acetate at pH 7.5, 150 mmol/L NaCl, 0.1% Tween, and 50 mmol/L magnesium acetate), the coated microtiter wells were filled with WBTH and incubated on ice for at least 20 min. This translation mixture was added to the prepared DES-coated microtiter wells. The plate was shaken for 1 h in a cold room. After three washes with ice-cold WBT, the retained ribosomal complexes were dissociated by two separate 10 min incubations in 100 µL EB (50 mmol/L Tris-acetate at pH 7.5, 150 mmol/L NaCl, 20 mmol EDTA, and 1 µL of RNasin inhibitor). In order to remove the DNA template, the reaction mixture was treated with 2 µL RNasin-free DNaseI (Promega, USA) for 15 min at 37°C.

### RT-PCR

The mRNA was isolated from the eluted solution using an RNeasy clean up kit (Qiagen, Germany) as described by the manufacturer. Selected mRNA was reverse transcribed to cDNA with the primer PM using a MLV reverse transcriptase (Invitrogen, USA). The obtained cDNA was amplified in a 20 µL PCR mixture for 35 cycles of 15 s at 95°C, 15 s at 56°C, and 30 min at 72°C with HF and LR using PfuUltra II Fusion HS DNA polymerase (Agilent, USA). Then the library was constructed by attaching the T7 promoter and the fPD using SOE PCR. The selected library could be inserted into the T-cloning vector for sequencing or subsequent cycles.

### The preparation of the DES-coupled magnetic beads

First, the DES with carboxyl was activated by carbodiimide. To 1 mL DMF, 3.68 mg DES, 2.87 mg EDC, 1.73 mg NHS were added and dissolved. The solution was stirred in the dark for 8 h at room temperature. The supernatant of active ester was obtained by centrifugation. Then, 500 µL (5 mg) magnetic beads were washed three times with 1 mL MES buffer (50 mM MES, pH 5.5) and resuspended in 625 µL MES buffer. Washing was achieved by using a magnet to pull the beads to the side of the tube, followed by aspiration of washing buffer. The above solution was slowly added into the magnetic beads solution under stirring. In order to react adequately, the mixture was stirred at 4°C for 12 h and then the linked magnetic beads were washed 3 times in 1 mL PBS with 0.1% Tween-20. Each wash included incubation with washing buffer for a minimum of 10 minutes. The DES-coupled magnetic beads were resuspended to a final concentration of 10 mg/mL in PBS with 0.01% Tween-20. The activity of the DES-coupled magnetic beads was examined by enzyme linked immunosorbent assay (ELISA).

### Affinity selection by magnetic beads

To select specific antibody fragments, 100 µL of 10 mg/mL DES-coupled magnetic beads were washed five times with ice-cold PBS and three times with ice-cold WBT, then stored at 4°C or −20°C. The preparation of the translation mixture is given in Section 2.6. The ice-cold beads were added to the mixture and incubated at 4°C for 60 min with gentle mixing. The liquid was removed and the EB was added to the beads. The following procedures were the same as above.

### Expression of soluble scFvs

After selection, dsDNAs were cloned into the vector pTIG-TRX for expression. Primers engineered with restriction sites *EcoRl* and *Xhol* were used to amplify the scFvs DNA. The PCR reaction was performed with PfuUltra II Fusion HS DNA polymerase (Agilent, USA) using 25 cycles of 94°C for 30 s, 55°C for 40 s, and 72°C for 1 min. The amplified scFvs DNA and pTIG-TRX vector were digested with *EcoRl* and *Xhol* and then purified using the Wizard SV GEL kit (Promega, USA). Ligations of prepared insert DNA and pTIG-TRX vector were carried out using T4 DNA ligase. The ligations were transformed into *E. coli* BL21 (DE3) and the soluble scFv proteins were expressed from each clone. In brief, single colonies were grown in 5 mL of LB medium with ampicillin (100 mg/mL) to an OD_600_ of 0.6 at 30°C/250 rpm, and induced by the addition of IPTG (final concentration 1 mM) for 4 h at 30°C/180 rpm. Each culture was centrifuged at 4000 rpm for 10 min. The cell pellet was resuspended in 2 mL of 1×PB (pH 7.4). The cells were ruptured by sonication and centrifuged at 10,000 rpm for 10 min. The supernatant was collected and tested for the presence of soluble scFv by Western blotting.

### Western blot analysis of scFvs

The His-tagged proteins were be detected by Western blotting using an Anti-His tag antibody. The scFvs concentrated by trichloroacetic acid (TCA) were separated by SDS-PAGE on 12% polyacrylamide gels. Pre-stained SDS-PAGE standards (Fermentas, USA) were used to calibrate protein molecular size. After SDS-PAGE, the proteins was transferred onto nitrocellulose membranes using a semidry electroblotter (Bio-Rad, USA). The transblotted membrane was blocked for 90 min at 37°C with PBSB (with 3% BSA) and then incubated for 1 h at 37°C with anti-His tag antibody (Zhongshan, China). A horseradish peroxidase (HRP)-labeled goat anti-mouse IgG was used to detect the bound anti-His tag antibody. The membrane was then incubated for 2 min at RT with a volume of DAB substrate solution sufficient to cover the membrane.

### Determination of binding activity to DES

To screen DES-specific scFvs, the expressed soluble scFvs were analyzed by indirect ELISA. Microtiter plates were coated with 100 µL of DES-ovalbumin (DES-OVA, 10 µg/mL in carbonate buffer solution) or OVA (10 µg/mL in carbonate buffer solution) as control overnight at 4°C. Plates were washed with washing buffer and the blocking buffer (1% w/v BSA in PBS, pH 7.4) was subsequently added at 37°C for 1 h with gentle shaking. Blocked plates were washed and 100 µL of expression supernatants at different dilutions were titrated followed by 2 h incubation at 37°C. ScFv binding was confirmed using a monoclonal mouse anti-His followed by goat anti-mouse antisera conjugated to HRP. Plates were developed with TMB-detection-solution and read at OD 450 nm.

Competition ELISA was performed to assess binding of antibodies to hapten. The crude scFv solution was preincubated with serial dilutions of soluble DES (0.0005–50 µg/mL). The antibody-competitor mix was then transferred to microtiter wells coated with 10 µg/mL OVA-conjugated DES and incubated for 2 h at 37°C. Unbound antibodies were washed with PBS supplemented with 0.05% Tween-20. Bound scFvs were detected as described above.

### Affinity analysis

The affinity of scFv against DES was assessed with SPR (Auto Lab, Netherlands). The purified DES-BSA (1 mg/mL) was immobilized on a sensor chip (Auto Lab, Netherlands) according to the supplier's protocol. DES-BSA was immobilized on a carboxytated sensor chip with standard amine coupling in sodium acetate buffer (pH 4.5). A range of concentrations of extracted scFv fragments were injected over the immobilized chip surface. The results were analyzed by Autolab ESPRIT Data Acquisition 4.3 and Kinetic Evaluation 5.0 software. The affinity of the selected scFvs was expressed by dissociation constants. The chip was regenerated by injection of repeated pulses of 100 mM HCl.

### Expression and purification of anti-DES scFv

The positive clones binding to immobilized DES were grown in 400 mL cultures and expressed scFv was extracted as described previously. Soluble scFv was purified by HisTrap HP metal affinity chromatography (GE Healthcare, USA) by following the manufacturer's instructions. The eluted scFv was tested by SDS-PAGE.
