# Correction: Selection of Diethylstilbestrol-Specific Single-Chain Antibodies from a Non-Immunized Mouse Ribosome Display Library

**DOI:** 10.1371/annotation/0cfd3d5f-c1d0-48f8-ad69-34a95e31a8d2

**Published:** 2012-07-10

**Authors:** Yanan Sun, Baoan Ning, Ming Liu, Xianjun Gao, Xianjun Fan, Jianqing Liu, Zhixian Gao

There was an error in the funding statement. The correct funding statement is: This work was supported by National Natural Science Foundation of China (Grant No.20907074, No.81030052) and Tianjin applied-basic & cutting-edge research program (09JCZDJC17700). The funders had no role in study design, data collection and analysis, decision to publish, or preparation of the manuscript. 

